# A Theoretical Study of the Hydration of Methane, from the Aqueous Solution to the sI Hydrate-Liquid Water-Gas Coexistence

**DOI:** 10.3390/ijms17060378

**Published:** 2016-05-26

**Authors:** Daniel Porfirio Luis, Alcione García-González, Humberto Saint-Martin

**Affiliations:** 1 CONACYT Research Fellow-Centro de Ingeniería y Desarrollo Industrial, Queréraro, Qro 76125, México; dpluisji@conacyt.mx; 2 Facultad de Ciencias Químicas, Universidad Autónoma de Nuevo León, Nuevo León 66451, México; alcione.garciagn@uanl.edu.mx; 3 Instituto de ciencias Físicas, Universidad Nacional Autónoma de México, Apartado Postal 48-3, Cuernavaca, Morelos 62251, México

**Keywords:** numerical simulations, analytical model potentials, hydrates, phase coexistence

## Abstract

Monte Carlo and molecular dynamics simulations were done with three recent water models TIP4P/2005 (Transferable Intermolecular Potential with 4 Points/2005), TIP4P/Ice (Transferable Intermolecular Potential with 4 Points/ Ice) and TIP4Q (Transferable Intermolecular Potential with 4 charges) combined with two models for methane: an all-atom one OPLS-AA (Optimal Parametrization for the Liquid State) and a united-atom one (UA); a correction for the C–O interaction was applied to the latter and used in a third set of simulations. The models were validated by comparison to experimental values of the free energy of hydration at 280, 300, 330 and 370 K, all under a pressure of 1 bar, and to the experimental radial distribution functions at 277, 283 and 291 K, under a pressure of 145 bar. Regardless of the combination rules used for *σ_C,O_*, good agreement was found, except when the correction to the UA model was applied. Thus, further simulations of the sI hydrate were performed with the united-atom model to compare the thermal expansivity to the experiment. A final set of simulations was done with the UA methane model and the three water models, to study the sI hydrate-liquid water-gas coexistence at 80, 230 and 400 bar. The melting temperatures were compared to the experimental values. The results show the need to perform simulations with various different models to attain a reliable and robust molecular image of the systems of interest.

## 1. Introduction

Gas hydrates are compounds formed by the inclusion of gas molecules in cavities of the crystal lattice of water, and they can exist at elevated pressures for temperatures somewhat above the melting point of hexagonal ice (ice Ih) . Depending on the properties of the guest gas molecules and the details of the hydrate formation procedure, different structures can be obtained, the most important being structure I (sI), structure II (sII) and structure H (sH) [1,2]. Typically, smaller gas molecules (such as methane, ethane and carbon dioxide) tend to form sI hydrates, while larger molecules preferentially form sII (propane, iso-butane) and sH (cyclohexane, cycloheptane) hydrates. These structures differ in the size of the cavities in the clathrate network of water molecules, as well as in the number of cavities of different types in the unit cell. The smallest cavity found in gas hydrates is the pentagonal dodecahedral cage (5^12^) comprising twelve pentagons (average radius < *r* > = 3.95 Å). Larger cavities include tetrakaidecahedral (5^12^6^2^, with < *r* > = 4.33 Å) and hexakaidecahedral (5^12^6^4^, with < *r* > ∼ 5 Å) cages, which can be found in sI and sII hydrates, respectively. The base of the notation designates the type of face, while the exponent the number of faces of the same type. The shapes and sizes of the cavities were first proposed by Claussen [3], who used a ball-and-stick model and searched for water aggregates that at the same time were capable of encaging a methane molecule and of accommodating into a space-filling crystal structure. The experimental corroboration was reported almost immediately [4] by Stackelberg and Müller and has been recently confirmed by high-resolution neutron diffraction [5] and by X-ray single-crystal analysis [6].

The formation of hydrates represents a problem for natural gas production, transportation and processing, because of possible water intake in the pipelines, especially in offshore fields. Different chemical inhibitors are available [7] to prevent the occlusion, which are classified as either thermodynamic or kinetic: in the former case, they alter the chemical potential of water in either the liquid or hydrate phase and thereby shift the boundaries on the phase diagram. Kinetic inhibition, on the other hand, is designed either to delay the initial nucleation or to alter the morphology of any crystals that do grow so as to ensure that they retain acceptable rheological properties. Alternatively, physical methods, such as the application of an electric field, can be used to prevent the accretion of the crystal by melting the incipient nucleation aggregates [8,9,10,11,12,13,14,15,16]. Furthermore, simulations of the process of hydrate decomposition at different cage occupancies have been studied by Myshakin *et al.* and English *et al.* [17,18]; they found that the decomposition rate depends sensitively on the hydration number. In another work, it was found that the dissociation of the hydrate is accelerated by the formation of methane bubbles, both in NaCl solutions and in pure water [19]. On the other hand, molecular simulations have been used to study the methane hydrate growth; Báez and Clancy [20] made one of the first contributions developing an hydrate-liquid distinction criteria when an hydrate crystal grows in a simulation. A remarkable advance was made by Walsh *et al.* [21] showing the spontaneous nucleation and growth of methane hydrate from a solution of methane and water; this was made possible by extending simulations into the microsecond domain. They used the TIP4P/Ice [22] water model and a united-atom methane model. Relative to the water models used in molecular simulations for the calculation of the melting point, Mastny *et al.* [23] have found good estimation for methane hydrate, while English and Clarke [24] for CO_2_, using potential models and interaction parameters that have been parameterized specifically for water-guest or hydrate systems. Molecular simulation has also been used to study water-methane interfaces or in the bulk aqueous phase to enhance our understanding of their thermodynamics properties [25]; this is important because nucleation would take place at or near the interface [1,26] or in the bulk aqueous phase [27].

Whereas the shapes and the number of water molecules of the gas-containing cavities in the crystal structures of gas hydrates are well established, the same is not true for the ordering of water molecules around non-polar solutes in aqueous solution. The deviations found for the entropies of vaporization of non-polar solutes in water, together with the large effects of temperature upon them, led to the idea that the water formed frozen patches or microscopic icebergs around such solute molecules, the extent of the iceberg increasing with the size of the solute molecule [28]. The success of Claussen’s prediction [3] of the clathrates seemed to substantiate the iceberg model of hydrophobic hydration, but it is now recognized that this extrapolation from the solid phase does not apply to the liquid phase, and the iceberg model has been discarded [29] on the basis of various results: from theoretical studies, the number of water molecules in the solvation shell of methane estimated from numerical simulations [30,31] ranges from *n_H_* = 16 to *n_H_* = 22; a calculation based on the number of water molecules in a spherical shell [32] surrounding methane yields *n_H_* = 14; and from an analysis of a large number of hydration shells obtained from numerical simulations [33], it was concluded that the probability of occurrence of a 5^12^ cage around methane in aqueous solution is much less than 10^−7^. From experimental studies, the integration of the C–O radial distribution functions (rdfs) obtained from neutron diffraction [34] yielded *n_H_* = 16, and no evidence was found that hydrophobic solutes enhanced the structure of water [35]; however, from a more recent comparison of ^13^C chemical shifts obtained from magic-angle-spinning nuclear magnetic resonance (MAS NMR) [36] for methane in the hydrate and in the aqueous phase, it was concluded that *n_H_* = 20, arguing that this value is indisputable, albeit with a dynamic aqueous methane hydration shell where water molecules might continuously enter and leave the hydration sphere. The same number is reported in a recent molecular dynamics study [33]. Nonetheless, the hydration number *n_H_* = 16 determined from neutron diffraction [34] still poses a problem of interpretation.

Theoretical studies of the methane-water systems complement the information gathered from experiments, by providing both interpretations at the molecular level and an inexpensive means to assess the feasibility and even the economic cost of using a certain method to impede hydrate formation. The reliability of the predictions obtained from numerical simulations depends on the accuracy of the molecular models that are employed. To be able to study the formation and the melting of hydrates, these models should ideally perform equally well over a range of thermodynamic conditions ample enough to comprise the three phases present in a pipeline: the gaseous methane, its aqueous solution and the crystalline solid. Unfortunately, this is currently out of the question: no water model exists to date that is capable of describing equally well the ices and the liquid. The best model for the ices, TIP4P/Ice [22], fails to reproduce the equation of state *ρ*(*T*) of the liquid, whereas TIP4P/2005 [37] is probably close to the best description of water that can be achieved with a non-polarizable model described with a single Lennard-Jones (LJ) site, and three charges though cannot reproduce the static dielectric constant *ϵ*(*T*) and produce a too-low melting temperature for ice Ih. This last feature is common to the more recent TIP4Q [38], which improved the agreement with experimental data of the liquid, especially the dielectric constant *ϵ*(*T*). Though the strongest interactions in hydrates are the same as those for ices, namely hydrogen bonding between water molecules, the size of the cages and, especially, their occupancy also depend on the gas-water interactions [39], methane in this case. The methane molecule can be modeled either considering all of the hydrogens, the all-atom approach (for instance the OPLS-AA [40]) or with an electrically-neutral single site, the united-atom (UA) approximation [30]; in both cases, the interaction with water has been modeled with a standard Lennard-Jones (LJ) potential. Whereas the non-zero charges of the AA approach were obtained from quantum calculations, the parameters of the LJ potential, *ϵ* and *σ*, were fitted to reproduce the methane-water interaction with a specific water model, the original TIP4P. The use of these methane models with different water models can be done either with combination rules or with a re-parametrization of the methane-water potential. This was the subject of a study by the group of Vega [41], who concluded that a 7% increase in the *ϵ_C,O_* parameter sufficed to reproduce the solubility of the gas and the properties of the methane hydrate, for the UA methane model combined with the TIP4P/2005 water model. However, in a more recent study of the three-phase coexistence, the same group used the TIP4P/Ice model with the original UA methane, but without the 7% correction [42]. Jensen *te al.* [43] and Michalis *et al.* [44] have also calculated this phase equilibrium line quite rigorously for methane hydrates directly from molecular simulation.

It becomes then relevant to compare the predictions of the different models on the behavior of the systems of interest, as this allows one to assess the robustness of the various conclusions that can be attained. Thus, the purpose of this study is to apply the different techniques of numerical simulations to compare the performance of the rigid models of water TIP4P/2005, TIP4P/Ice and TIP4Q in reproducing the experimental data of the hydration of methane. Therefore, in the present work, we present the results of Monte Carlo (MC) and molecular dynamics (MD) simulations of the diluted aqueous solution of methane, the sI hydrate and the methane gas-liquid water-sI hydrate coexistence, performed with the three previously-mentioned water models, combined with the OPLS-AA all-atom model for methane [40] and a more recent united-atom (UA) model [41]. The comparison to experimental data is made with the hydration free energies, the coordination properties, the sI hydrate thermal expansivity and the gas-liquid-hydrate coexistence conditions.

## 2. Results and Discussion

### 2.1. Free Energies of Hydration

A minimum requirement for an empirical model intended to correctly describe the interaction of methane with water is the reproduction of the hydration free energy Δ*hyd^G^* at infinite dilution, ideally at various different temperatures [41]. The very low solubility of hydrophobic molecules, a ratio of about 1/4000 waters under ambient conditions [45], poses a problem for simulations with a much smaller number of water molecules, in this case, the ratio being 1/241. However, the very low energy of the methane molecule with water, and even lower with the other methane molecule, allows one to obtain quantitative agreement with experiments from the higher simulated concentration.

**Figure 1:**
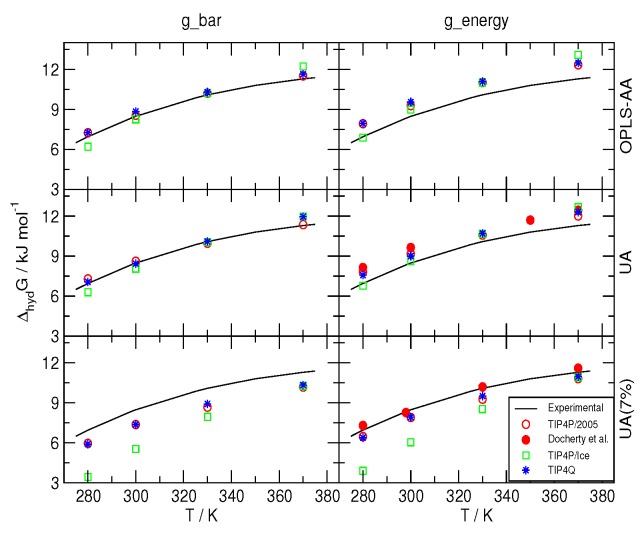
Comparison to the experimentally determined hydration free energy of methane (as reported in Reference [41]) of those obtained from MD simulations with Bennett’s Acceptance Ratio [46] (g_bar) and evenly spaced (g_energy) thermodynamic integration. OPLS-AA: All-atom force-field from Reference [40]; UA: United-atom model from Reference [41]; UA (7%): Same model with a 7% correction for the C−Øinteraction. The symbols were given sizes slightly larger than the corresponding standard deviations.

The hydration free energy Δ*hyd^G^* was computed as described in Section 3 for the model combinations in Table 1, and the results are depicted in Figure 1, along with those reported in [41] for the experimental data and the values for combinations 2005-2 and 2005-3. It can be seen that when the Bennett’s Acceptance Ratio [46] (BAR) was used, the combinations 2005-1, 2005-2, Q-1 and Q-2 yielded values in very close agreement with the experiment, whereas the TIP4P/Ice water model produced somewhat larger deviations. With this method, all of the estimates for Δ*hyd^G^* with the 7% correction on the C−O interaction were underestimated, with TIP4P/Ice increasing the discrepancy at lower temperatures. As it turns out from using the BAR method, the 7% correction worsens the agreement with experimental data; thus, the conclusion is opposite that in [41]. However, the computation of Δ*hyd^G^* with equal acceptance resulted in agreement with the data that were obtained from MC with the Widom insertion method [47] in [41]. These differences highlight a common problem of all empirical molecular models, *viz*. the dependence of the parametrization on the methods used to compute the target experimental data. It is worth mentioning that, to the best of our knowledge, none of the more recent simulations on the formation and the melting of hydrates [42,43,48,49,50] employs any correction to the so-called Lorentz–Berthelot combination rules.

### 2.2. Radial Distribution Functions and Coordination Numbers n_H_

In the present work, Monte Carlo simulations were performed on a system with one methane molecule in 343 water molecules, which amounts to an order of magnitude larger than the solubility of methane [45]. The sampling was done on the isothermal-isobaric (NpT) ensemble, using isotropic pressure, the analytical model potentials and the thermodynamic conditions that are described in Section 3. A spherical cutoff of 1 nm was used, and long-range interactions were handled with Ewald sums. One MC step comprised 5000 trials, divided into the following fractions: 0.003 for CH_4_ moves, 0.994 for H_2_O moves and 0.003 for attempts to change the volume. The molecular displacements and rotations, as well as the volume changes, were adjusted to yield a 50% acceptance: trial ratio. The simulation of each system started from an arbitrary configuration, and an initial run of 3 × 10^4^ MC steps was used for equilibration. Production runs comprised 7 × 10^4^ MC steps, and their statistical significance was assessed with the blocking method [51], whence an average and a standard deviation were assigned, for instance, to the densities. The standard deviation was the same for all runs, ±2 kg·m^−3^. The TIP4P/Ice model systematically produced lower densities, albeit only slightly (no more than 1%).

**Figure 2:**
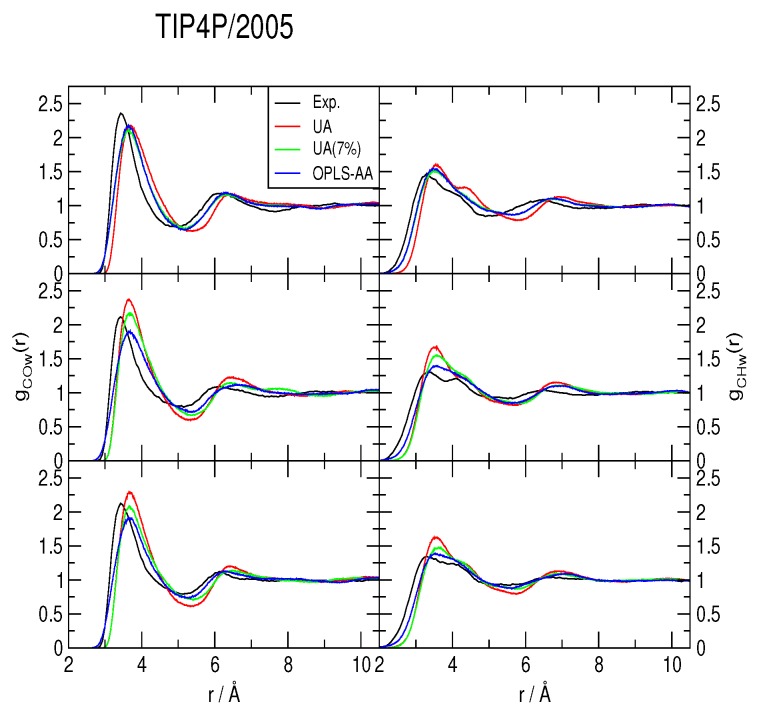
Comparison to the experimentally determined [34] methane-water radial distribution functions of those obtained from simulations with the TIP4P/2005 water model at 277 K (**top**), 283 K (**middle**) and 291 K (**bottom**), all under a pressure of 145 bar. UA: United-atom model from Reference [41]; UA (7%): Same model with a 7% correction for the C−O interaction; OPLS-AA: All-atom force-field from Reference [40].

**Figure 3:**
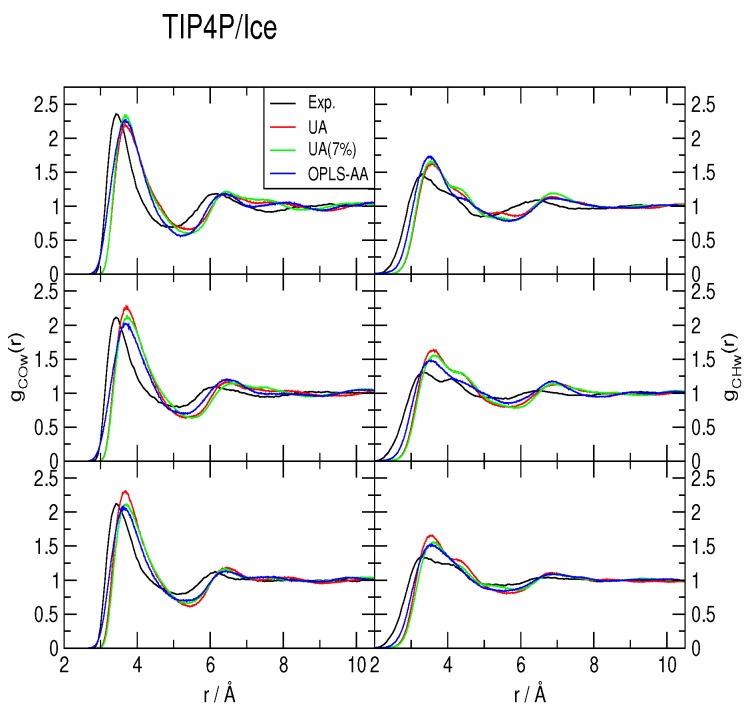
Comparison to the experimentally determined [34] methane-water radial distribution functions of those obtained from simulations with the TIP4P/Ice water model at 277 K (**top**), 283 K (**middle**) and 291 K (**bottom**), all under a pressure of 145 bar. UA: United-atom model from Reference [41]; UA (7%): Same model with a 7% correction for the C−O interaction; OPLS-AA: All-atom force-field from Reference [40].

**Figure 4:**
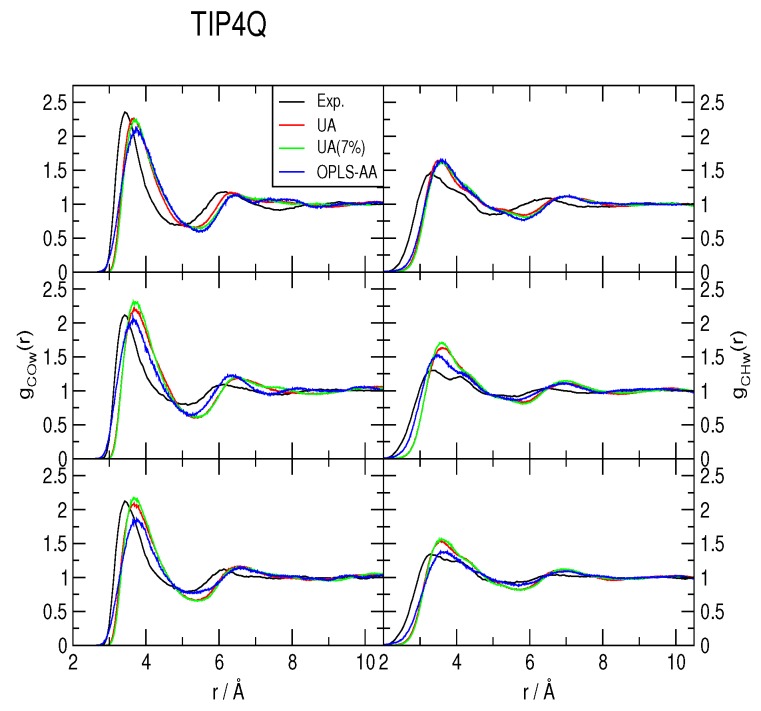
Comparison to the experimentally determined [34] methane-water radial distribution functions of those obtained from simulations with the TIP4Q water model at 277 K (**top**), 283 K (**middle**) and 291 K (**bottom**), all under a pressure of 145 bar. UA: United-atom model from Reference [41]; UA (7%): Same model with a 7% correction for the C−O interaction; OPLS-AA: All-atom force-field from Reference [40].

The methane-water rdfs *g_COw_*(*r*) and *g_CHw_*(*r*) are shown in Figure 2, Figure 3 and Figure 4, for the TIP4P/2005, TIP4P/Ice and TIP4Q water models, respectively. All models predict a more ample cavity for methane, which is inferred from the *ca*. 0.25 Å shift to the right of the first peak in both rdfs. In general, the OPLS-AA produced less structure than the UA model. While the 7% correction to *ϵ_C,O_* flattened the rdfs when used with both TIP4P/2005 and TIP4P/Ice, it had a much smaller (and opposite) effect with TIP4Q. In fact, it can be noticed in Figure 4 that TIP4Q is less sensitive to the choice of CH_4_ model.

Because the first minimum of the rdfs does not attain zero in any case, there is not a clear-cut first hydration shell; nonetheless, all of the *g_COw_*(*r*) first minima occur around *r* = 5.5 Å. Hence, instead of integrating *g_COw_*(*r*), a histogram was made with the number of water molecules at a maximum distance of *r* = 5.5 Å, sampled each MC step, to estimate the coordination number *n_H_*. All histograms turned out to be normal distributions. All of the simulations yield *n_H_* ∼ 20 ± 3, in agreement with the MAS NMR data of [34], but with distributions that range from *n_H_* = 11 to *n_H_* = 30; that is to say very dynamic.

### 2.3. The sI Hydrate

**Figure 5:**
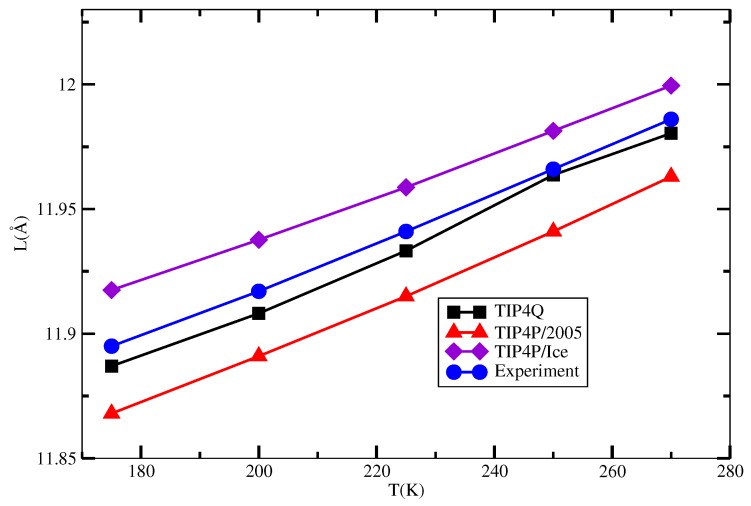
Unit lattice length as a function of temperature for the combinations 2005-2, Q-2 and Ice-2 in Table 1 and experimental data.

The behavior of the unit cell length of the sI hydrate as a function of temperature that resulted from the simulations described in Section 3 is depicted in Figure 5, and the numerical values are presented in Table 2. It can be seen that the TIP4Q gives the best agreement with the experimental values, whereas TIP4P/2005 has the correct trend, but slightly underestimated in some 0.03 Å. On the other hand, the TIP4P/Ice overestimated the experimental values in some 0.02 Å.

### 2.4. The Gas-Liquid-Hydrate Coexistence

This three-phase coexistence has already been studied with different molecular models [42,43,44,48,50]. Because TIP4P/Ice yields the best reported reproduction of the phase diagram of the ices [22], the combination Ice-2 in Table 1 was used in [42,43,50], but somewhat different results were obtained, which have been ascribed to the different area of the contact surfaces. This discrepancies corroborate the observation made in Section 2.1 about the dependence on the simulation methods used to compute the target values, of the parametrizations of empirical molecular models, thus supporting the main idea of the present work, that different models have to be used to attain reliable molecular images of the systems of interest. Thus, the two water models TIP4P/2005 and TIP4P/Ice are used in this work for a comparison with those previous studies, and the TIP4Q potential is added to check on its performance, all combined with the UA model methane.

**Figure 6:**
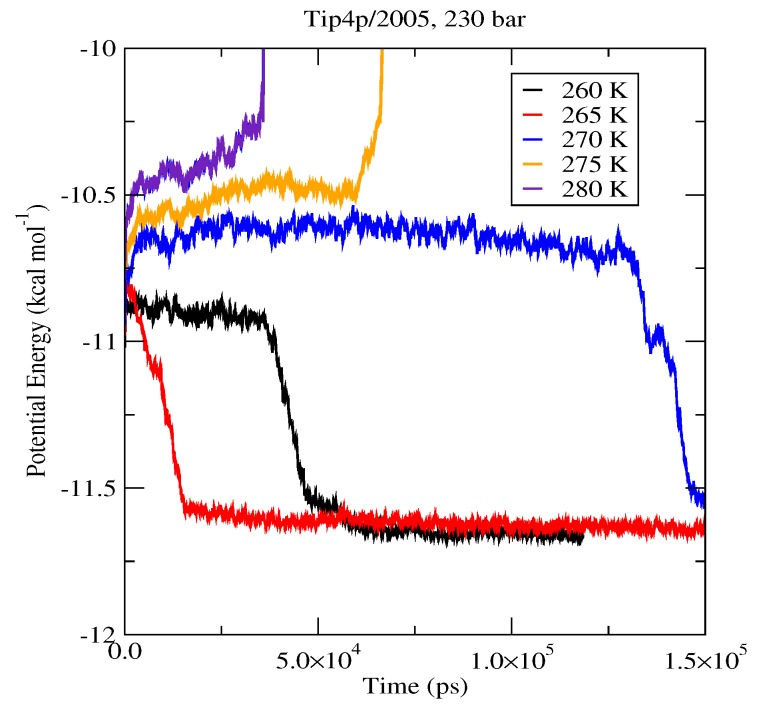
Evolution in time of the potential energy in simulations with the TIP4P/2005 model of the system in Figure 12.

**Figure 7:**
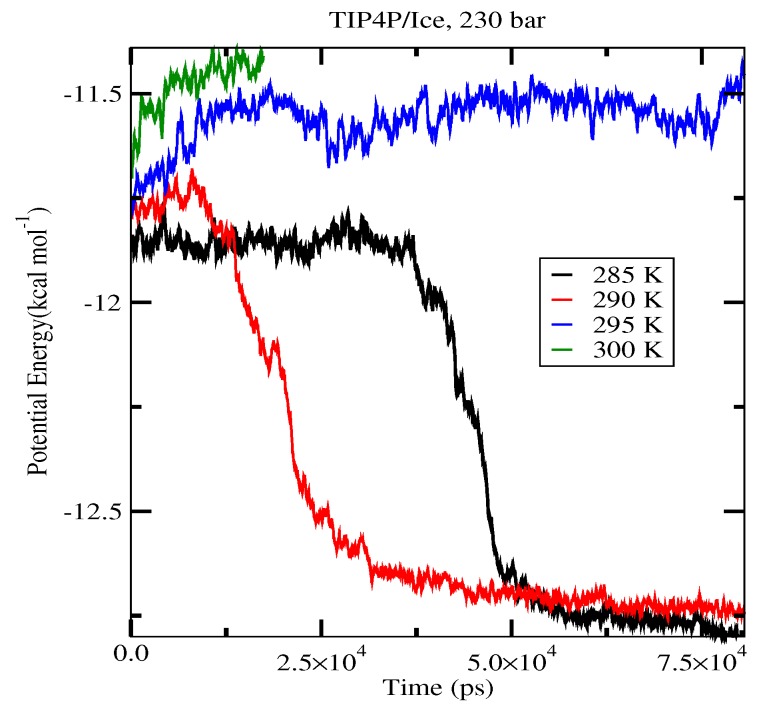
Evolution in time of the potential energy in simulations with the TIP4P/Ice model of the system in Figure 12.

**Figure 8:**
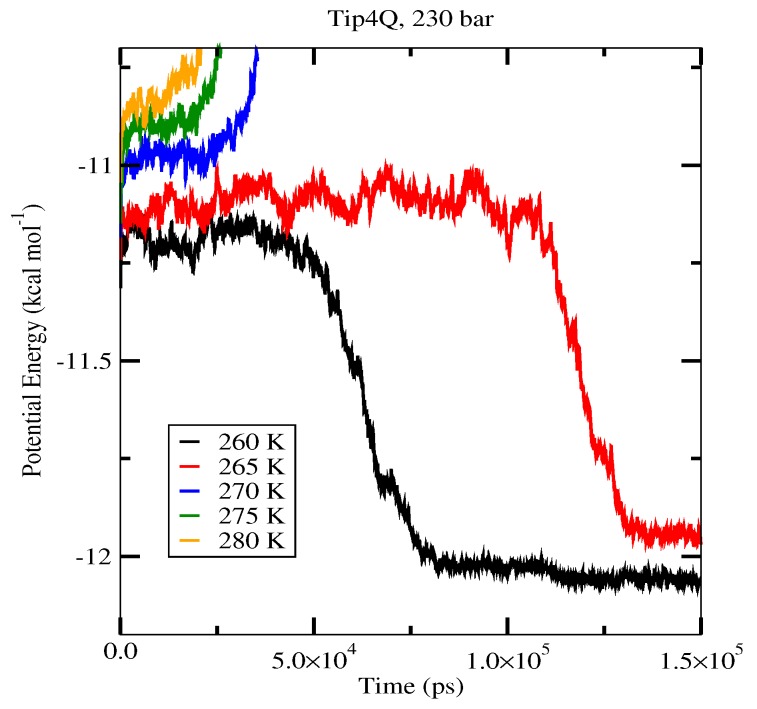
Evolution in time of the potential energy in simulations with the TIP4Q model of the system in Figure 12.

**Figure 9:**
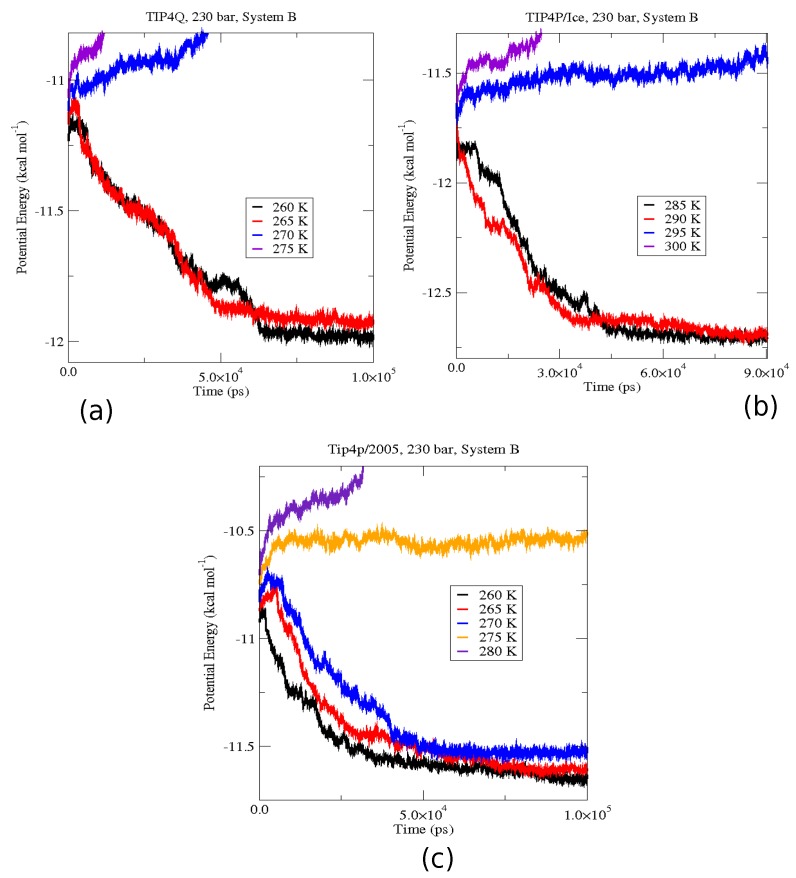
Evolution of the potential energy at a fixed pressure of 230 bar for system B. (**a**) Shows the results for TIP4Q; the three-phases coexistence temperature calculated was 267.5 K (+/−2.5 K); (**b**) Shows the results for TIP4P/Ice; the three-phases coexistence temperature calculated was 292.5 K (+/−2.5 K); (**c**) Shows the results for TIP4P/2005; the three-phases coexistence temperature calculated was 272.5 K (+/−2.5 K).

**Figure 10:**
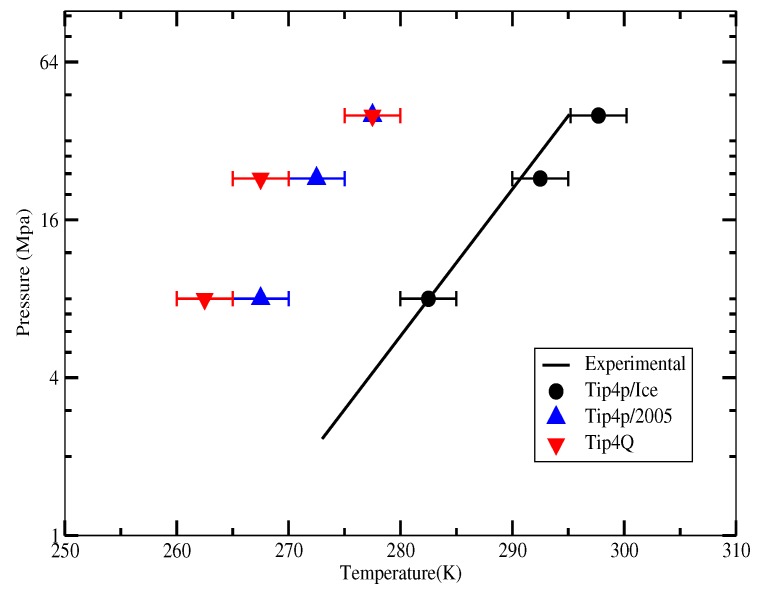
Three-phase coexistence temperature at different pressures with different water models, experimental results were taken from [1].

**Figure 11:**
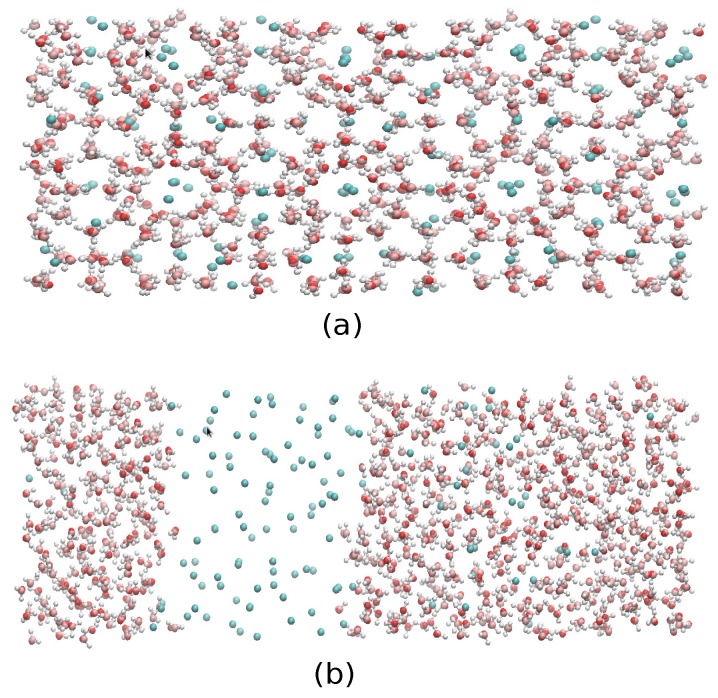
Final configurations of system A at *P* = 230 bar and two temperatures, (**a**) *T* = 290 K and (**b**) *T* = 300 K, where it can be seen that the former resulted in complete formation of the hydrate, whereas the latter yielded a liquid-like geometry.

The first set of simulations was made at 230 bar, and the evolution in time of the potential energy at various different temperatures is shown in Figure 6 for TIP4P/2005; in Figure 7, for TIP4P/Ice, and in Figure 8, for TIP4Q, all with System A. In Figure 9, we show the results for the three models, all with System B. The resulting three-phase coexistence temperatures, T_3_, are presented in Table 3 and in Figure 10; a good agreement was found with the data in [42,44] for the TIP4P/2005 and TIP4P/Ice models, and the value obtained for TIP4Q is close to that of TIP4P/2005. It can be seen that the three water models give the same coexistence temperature for System A and for System B at 230 bar, with the difference that System B crystallizes faster than System A with the same water potential. For example, using the TIP4Q water model, System B crystallizes in around 50,000 ps, while System A crystallizes in 150,000 ps. With the TIP4P/Ice water model, System B crystallizes in around 40,000 ps, while System A in 50,000 ps. Additionally, with the TIP4P/2005, System B crystallizes in around 40,000 ps, while System A in 150,000 ps. Opposite the findings in [52], the simulation of a larger system affected solely the rate at which crystallization occurred, but not the predicted coexistence temperature. It can be seen that both the TIP4Q and TIP4P/2005 models underestimate the experimental values of T_3_ for all pressures. This is more clearly seen in Figure 11, where the logarithm of the pressure has been plotted as a function of temperature. The experimental data have been taken from [1]. The TIP4P/Ice water model is the best of the three models considered in this study, in reproducing the coexistence temperature for the range of pressures studied in this work. The direct coexistence method possesses an inherent degree of stochasticity [44,53], which is why our result of the TIP4P/Ice model at 400 bar is slightly different from the results of Conde and Vega (−5 K) and Michalis *et al.* (+4.1 K). Additionally, for the TIP4P/2005 model at 400 bar, our result is also slightly different from the result of Conde and Vega (+1.5 K). The graphs of the simulations under pressure of 80 bar and 400 bar are not shown.

The method used in this work to determine T_3_ has been criticized [54] because it considers melting as an isothermal process, whereas it is more closely adiabatic in the real system, with significant spatial and temperature gradients. While taking them into account does modify the rate and the mechanism of decomposition [24,52,54], no critical effect on T_3_ has been reported [70].

## 3. Methods

In the simulations in this study, we used the rigid/non-polarizable TIP4P/Ice, TIP4P/2005 and TIP4Q water potentials. The TIP4P/Ice and TIP4P/2005 water models have an LJ interaction site located on the oxygen atom, positive charges located at the positions of the H atoms and a negative charge located at a distance *d_OM_* from the oxygen along the H–O–H bisector; whereas the TIP4Q water model has an LJ interaction site located on the oxygen atom, positive charges located at the positions of the H atoms, a positive charge located at the position of the O atom and a negative charge located at a distance *d_OM_* from the oxygen along the H–O–H bisector. The parameters of all of the molecular models used in this work are shown in Table 4 and Table 5. When the OPLS-AA model was used for methane, the Lennard–Jones parameters for the C−O and H−O interactions resulted from the following geometric averages:

$$\begin{array}{c} \epsilon_{i,j}=\sqrt{\epsilon_{i,i}\epsilon_{j,j}}\\ \sigma_{i,j}=\sqrt{\sigma_{i,i}\sigma_{j,j}} \end{array}$$

which is the default combination for the OPLS-AA force-field, whereas with the UA methane, the arithmetic mean was used for $\sigma_{i,j}=\frac{1}{2}\left(\sigma_{i,i}+\sigma_{j,j}\right)$. Furthermore, the 7% correction recommended in [41] was also applied to *ϵ_C,O_*, thus yielding nine model combinations that will henceforth be referred to as labeled in Table 1.

The GROMACS 4.5.1 package [55,56,57,58] was used for MD simulations to compute the hydration free energy of methane with the thermodynamic integration method [46,59,60,61,62] built in it. A system with one methane molecule in 241 water molecules was simulated under constant pressure and temperature (NpT ensemble), at 1 bar, controlled with the Berendsen barostat and at temperatures of 280, 300, 330 and 370 K, controlled by using the stochastic dynamics (sd) integrator with a 2-fs time-step, as described in the GROMACS user manual [63] (for the thermodynamic integration and BAR [46] methods, see Section 3.12.2 in user manual).

A homemade Monte Carlo program was used with a system of one methane molecule in 343 water molecules, to simulate the dilute aqueous solution of methane under a pressure of 145 bar and at 277, 283 and 291 K, to obtain the coordination numbers *n_H_* and the radial distribution functions (rdfs) and to compare them to the experimentally-determined data under the same conditions [34,36]. This was done because in Monte Carlo, the pressure and temperature controls are exact, and the structural information can be obtained somewhat more readily.

The GROMACS 4.5.1 code was also used to simulate the fully-occupied sI hydrate in the NpT ensemble: the unit cell, with a side of length 1.203 nm, was built according to the X-ray crystallographic data [64]; the hydrogen atoms of the water molecules were distributed randomly, but following the Bernal-Fowler rules and changing the orientations until achieving a near-zero total dipole moment. The unit cell was then replicated 2 times in each orthogonal direction (2 × 2 × 2) to form a cubic cell of side 2.406 nm, constituted by 368 molecules of water and 64 of methane. The pressure was kept at 30 bar by means of the isotropic Parrinello-Rahman barostat [65,66] with time constant for coupling tau_p = 2, and the temperature was successively fixed at 175, 200, 225, 250 and 270 K with a Nosé-Hoover thermostat [67,68]. For the long-range Coulombic interaction, the Particle Mesh Ewald (PME) algorithm was used with a cut-off radius of 0.9 nm; an LJ interaction was implemented with a cut-off radius of 0.9 nm; and the Lorentz-Berthelot mixing rules were implemented in both cases. The simulations were implemented as NpT MD simulations using three-dimensional periodic boundary conditions. The system was run for 200 ps at each temperature, with a 3-fs time-step, to compute the thermal expansivity of the sI hydrate model. This time span was proven to yield statistically-meaningful averages for the unit cell length in [41].

**Figure 12:**
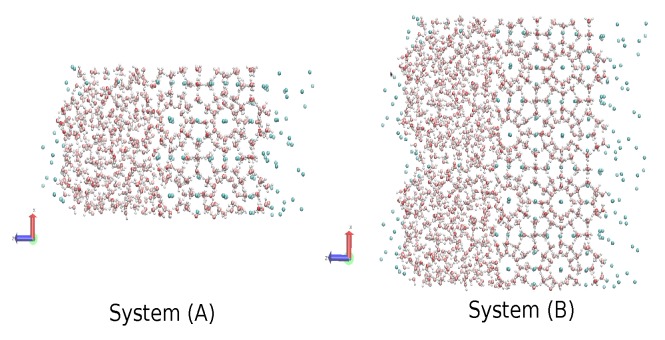
Systems used as seed for all the simulations of the methane-liquid water-sI hydrate, red arrow indicates x-axis and blue arrow indicates *z-axis.*

The same MD code with the direct phase coexistence method [69] was used to calculate the three-phase gas-liquid-hydrate coexistence temperature. We used the same time-step, the same thermostat and the same barostat, but allowing each of the three orthogonal directions to fluctuate independently, as described in [42]. It is worth mentioning that this procedure has been acknowledged to lead to an appropriate computational prediction of the phase diagram [70]. We made the coexistence analysis on two systems; System A comprised the same initial sI hydrate in contact with two other cubic boxes of the same size, one with 368 waters in the liquid phase to one side and another with 64 methanes in the gas phase to the other side, that yielded a computational cell of size 2.406 nm × 2.406 nm × 7.218 nm, with the contact interfaces perpendicular to the *Z*-axis. Additionally, System B was comprised of System A replicated 2 × 1 × 1 times; this means that the hydrate phase of System B comprised 736 water molecules and 128 methane molecules; the liquid phase comprised 736 water molecules; and the gaseous phase comprised 128 methane molecules; which yielded a computational cell of size 4.812 nm × 2.406 nm × 7.218 nm (Figure 12). To equilibrate the initial configurations with each model, a short 50-ps simulation was performed at 250 K under pressures of 80, 230 and 400 bar for System A and 230 bar for System B, which did not result in either melting or crystallization. Temperature scans were then performed at 80, 230 and at 400 bar for System A and 230 bar for System B, using the same controls and time-step for all simulations of each model. The systems were simulated at each temperature while analyzing the evolution of the potential energy as a function of time. The increase in potential energy indicated melting, whereas its decrease indicated crystallization. The equilibrium phase coexistence temperature was taken as an average of the lowest temperature at which the hydrate melted and the highest temperature at which the system froze, as in [42,48].

## 4. Conclusions

A series of MC and MD simulations were performed to calibrate the predictions that can be obtained from different models on the hydration of methane. Whereas TIP4P/Ice has a better performance for the ice phase diagram, TIP4P/2005 and TIP4Q provide a better description of liquid water; thus, it was no surprise that these two latter produced better agreement with the experimental data of the diluted aqueous solution of methane. The hydration free energy Δ*hyd^G^* was computed in this work with a more robust algorithm than in previous studies, which resulted in agreement with experimental data for all six combinations of water-methane models with their original parameters, regardless of the combination rule used for *σ_C,O_*, opposite the conclusion in [41].

Finally, we have performed molecular dynamics simulations to estimate the three-phase (methane hydrate-water-methane) coexistence temperature T_3_ at three different pressures (80, 230 and 400 bar) by using the direct coexistence method and the three water models (TIP4P/Ice, TIP4Q and TIP4P/2005) with the UA model methane. The results showed that the three-phase coexistence temperatures obtained with the TIP4P/Ice model were in good agreement with the experimental data. Results obtained by TIP4P/2005 and TIP4Q models were shifted to lower temperatures by about 20 and 25 K, respectively, with respect to the experimental data. A caveat is in order, as the method used to determine the coexistence temperatures relies solely on the behavior of the potential energy, disregarding other criteria to track the formation/melting of the hydrate. To be on the safe side, the final configurations of System A at *P* = 230 bar and two temperatures, *T* = 290 K and *T* = 300 K, are shown in Figure 12, where it can be seen that the former resulted in the complete formation of the hydrate, whereas the latter yielded a liquid-like geometry. We have observed that System A and System B give the same coexistence temperature and that System B crystallized faster than System A using the same potential and the same thermodynamics conditions; this could mean that using the direct coexistence method with bigger systems could result in better and faster results. The unexpected better performance of TIP4Q with regard to the thermal expansivity of the fully-occupied sI hydrate in the vicinity of the melting conditions suggests that the ability of TIP4P/Ice to produce a higher melting temperature of the hydrate, and perhaps also of ices, is related to the lower density of the model.

## Figures and Tables

**Table 1:** Combinations of water and methane models used in this work.

Water Model	OPLS-AA	UA	Corrected UA
TIP4P/2005	2005-1	2005-2	2005-3
TIP4P/Ice	Ice-1	Ice-2	Ice-3
TIP4Q	Q-1	Q-2	Q-3

**Table 2:** Unit cell length (in Å) as a function of temperature, under a pressure of 30 bar. The values for TIP4P/2005 are taken from Reference [41].

T/K	TIP4Q	TIP4P/2005	TIP4P/Ice	Expt.
175	11.887	11.868	11.917	11.895
200	11.908	11.891	11.937	11.917
225	11.933	11.915	11.958	11.941
250	11.964	11.941	11.981	11.966
270	11.980	11.963	11.999	11.986

**Table 3:** Three-phase coexistence temperature (T_3_/K) determined for the three water models at different pressures.

Pressure/Bar.	TIP4Q	TIP4P/2005	TIP4P/Ice
80 (System A)	262.5 K	267.5 K	282.5 K
230 (System A)	267.5 K	272.5 K	292.5 K
230 (System B)	267.5 K	272.5 K	292.5 K
400 (System A)	277.5 K	277.5 K	297.5 K

**Table 4:** Parameters of the water models used in this work. *R* = 8.31451 J·mol^−1^·K^−1^ is the molar gas constant.

Model	*σ*/Å	(*ϵ*/*R*)/K	*q_H_*/*e*	*q_O_*/*e*	*l_OM_*/Å
TIP4P/2005	3.1589	93.2	0.5564	0.0	0.1546
TIP4P/Ice	3.1668	106.1	0.5897	0.0	0.1577
TIP4Q	3.1666	93.2	0.5250	0.5	0.0690

**Table 5:** Parameters of the methane models used in this work. *R* = 8.31451 J·mol^−1^·K^−1^ is the molar gas constant.

Model	*σ*/Å	(*ϵ*/*R*)/K	*q*/*e*	*r_CH_*/Å
OPLS-AA C	3.50	33.2123	−0.240	1.094760
OPLS-AA H	2.50	15.0965	0.060	
UA CH_4_	3.73	147.5	0.0	
